# Social determinants of oral health in migrants at the Spanish border

**DOI:** 10.3389/fpubh.2025.1641311

**Published:** 2025-08-01

**Authors:** Juan Martín Hernández, Ignacio Barbero Navarro, Diego Rodríguez Menacho, Paloma Villalva Hernández, Jose María Barrera Mora, David Ribas-Pérez, Antonio Castaño Séiquer

**Affiliations:** ^1^Servicio Andaluz de Salud, Spain; ^2^Department of Stomatology, Universidad de Sevilla, Sevilla, Spain

**Keywords:** oral health, health inequities, migrant populations, epidemiology, Melilla, Spain

## Abstract

**Background:**

Health equity, particularly in oral health, remains a challenge for socially excluded populations such as migrants. This study investigates the oral health status and associated social determinants of health among adult immigrants residing at the Temporary Stay Center for Immigrants (CETI) in Melilla, Spain.

**Methods:**

A cross-sectional study was conducted in March 2024 involving 128 adult CETI residents. Data collection included standardized oral examinations following WHO guidelines and structured questionnaires assessing sociodemographic variables, health habits, and dental care history. Statistical analyses were performed using SPSS 29.0, with significance set at *p* < 0.05.

**Results:**

The sample was predominantly Latin American (80.5%) and male (67.2%), with a mean age of 34.85 years. While most participants reported good oral hygiene habits, 67.2% were partially edentulous, and the mean Decayed, Missing, Filled, Teeth (DMFT) index was 9.73—higher than national averages. Only 9.4% used dental prostheses. Significant differences were observed between Latin American and African subgroups regarding age distribution, oral hygiene habits, and access to dental care.

**Conclusion:**

Migrant populations at CETI face substantial oral health challenges and disparities linked to origin, education, and access to care. Ongoing epidemiological monitoring is essential to inform tailored, equity-oriented public health interventions that address the dynamic needs of these populations.

## 1 Introduction

One of our main responsibilities as healthcare professionals is to ensure equity in access to healthcare services and treatments aimed at improving patients’ overall health. But what exactly is health equity according to the World Health Organization (WHO)? It is defined as:

“The absence of unfair and avoidable or remediable differences in health among population groups defined socially, economically, demographically or geographically” (1).

As indicated by this definition, key drivers of health equity include social, economic, demographic, and geographic conditions. These factors tend to have a greater impact on disadvantaged groups, highlighting the importance of focusing research efforts on these populations to effectively address and reduce health inequities in comparison to the broader population.

Health inequalities are significantly shaped by what the WHO calls the social determinants of health (SDH). These are non-medical factors that influence health outcomes—broadly encompassing the conditions in which people are born, grow, live, work, and age. These conditions are shaped by the distribution of money, power, and resources at global, national, and local levels, including political systems, social norms, and economic policies. Health follows a social gradient—those with lower socioeconomic status generally experience worse health outcomes (2). Migrant populations are particularly vulnerable, as they are often exposed to multiple risk factors simultaneously.

It is incorrect to assume that social exclusion only occurs in developing countries. Even in high-income nations, there are socially excluded groups. For instance, Harvard University in the United States has implemented social dentistry programs aimed at low-income families, homeless individuals (3, 4), Spanish-speaking minorities (5, 6), and people with disabilities (7). In Spain, similar initiatives are conducted by the Universities of Valencia and Seville, which have developed specific programs for vulnerable populations (8, 9).

Building on this, it is important to recognize that these same social dynamics influence not only general health but also oral health, which is the specific focus of our work. Scientific evidence reveals a strong correlation between social inequalities and poor oral health, making oral health a valuable indicator of how social disadvantages impact overall well-being (2, 8–11).

Moreover, oral health plays a crucial role in daily functioning, nutrition, speech, and self-esteem. It can also serve as a gateway for detecting systemic diseases and chronic conditions. As such, poor oral health reflects and exacerbates broader health inequalities, underlining the need for targeted interventions in disadvantaged communities.

Achieving equity in oral health requires more than knowledge of social inequalities—it also demands an understanding of individuals’ life histories and societal contexts. In the case of migrants, this includes both their country of origin and the host society in which they aim to integrate.

In recent years, numerous preventive social dentistry programs have been developed for marginalized groups, aligned with the WHO’s holistic definition of health as a state of complete biological and psychological well-being influenced by sociocultural determinants (2).

Before implementing preventive actions aimed at immigrants and refugees, it is essential to assess their current health status and determine the level of intervention needed to improve it. With an initial epidemiological survey that would provide a deeper understanding of the target population, not only in terms of objective oral health status but also regarding relevant social, personal, and geographic determinants.

Migration is an increasingly important global issue that is significantly influencing the course of the 21st century. Migrants often face vulnerable and unstable circumstances as they seek improved living conditions in countries with unfamiliar cultures, languages, and social structures—many entering via irregular routes. Consequently, migration presents complex challenges related to personal well-being, humanitarian concerns, and social exclusion (12). The magnitude of this issue is underscored by growing global economic disparities and ongoing conflicts. The emotional and psychological toll of extreme migration experiences has even led to the identification of the so-called “Ulysses Syndrome,” a stress-related condition affecting many migrants (13).

Epidemiological studies serve as the primary source of information on disease distribution, health determinants, and risk factors within populations, and are vital for the design of effective preventive and therapeutic programs (14).

Each migrant population is unique and must be studied accordingly. Individual backgrounds, personal histories, and identified SDH factors (3) should be taken into account. It is crucial to understand how each specific population arrives in the host country, where they come from, what language they speak, their education level, and other contextual elements that affect their physical and psychological health, integration capacity, and access to healthcare systems. Facilitating access to healthcare services is now recognized as one of the most important factors in improving the health of migrant populations (15, 16).

It is also necessary to evaluate the host country’s healthcare system—its strengths, limitations, and ability to integrate marginalized populations—to provide the best possible support to incoming migrants (17–19).

In our case, the immigrant population under study is housed at the Temporary Stay Center for Immigrants (CETI) in the Autonomous City of Melilla, Spain. Unlike other autonomous regions in Spain, Melilla does not conduct regular epidemiological surveys on oral health—an important tool for shaping public health policy. These surveys are key to achieving national oral health goals set by the General Council of Dentists of Spain for (18), as well as global objectives outlined by the WHO (2).

Acknowledging this data gap, we have conducted several epidemiological studies in Melilla—some focused on local health conditions (20) and most targeting the immigrant population at CETI (21–23).

Our ongoing research has revealed that the CETI migrant population is highly dynamic. While it was once composed mainly of sub-Saharan and North African migrants (as one might expect due to Melilla’s geographical location), the current population is predominantly Latin American. These groups differ significantly in language, health habits, and needs. Understanding such shifts requires knowledge of local and national political and migration policies, which we will discuss later in this work. Shifts in migratory flows in recent years, along with the relocation of existing migrant populations to other regions of Spain or Europe, may account for the observed changes in predominant nationalities (24).

This variability underscores the necessity of conducting this cross-sectional epidemiological study, which aims to inform and improve public health interventions tailored to the changing needs of CETI’s population. Well-targeted oral health education programs have been shown to be particularly effective for refugees (16).

The main objective of this study was to assess the oral health status of socially excluded adult immigrants living at the CETI in Melilla, and to provide data that will help policymakers develop informed, effective public health strategies.

## 2 Materials and methods

### 2.1 Study design

This study employed a cross-sectional design. Data were collected through a survey administered to 128 adult residents at the Temporary Stay Center for Immigrants (CETI) during the last week of March 2024. The methodology included a standardized oral cavity examination in accordance with World Health Organization (WHO) guidelines. Additionally, a structured questionnaire focusing on oral health was used. This questionnaire is validated by the WHO and included in the manual *Oral health surveys: Basic methods* (25). Specifically, Annexes 1 and 7 of the manual were utilized as we can see as an Supplementary Table 1.

### 2.2 Population characteristics

#### 2.2.1 Gender

The study sample included 128 individuals, of whom 86 (67.2%) were men and 42 (32.8%) were women. Despite the numerical difference, the gender differences—even when analyzed by age groups—were not statistically significant. The Chi-square test for gender distribution between the two ethnic groups evaluated also showed no significant difference.

#### 2.2.2 Age

Participants ranged in age from 18 to 66 years, with a mean age of 34.85 years. For statistical purposes, age was grouped into three categories: Young Adult (18–29 years; *n* = 47, 36.7%), Adult (30–50 years; *n* = 68, 53.1%), and Older Adult (51–66 years; *n* = 13, 10.2%).

#### 2.2.3 Origin

Subjects came from diverse countries: Chile (*n* = 1), Colombia (*n* = 55), Cuba (*n* = 1), Honduras (*n* = 2), Mali (*n* = 4), Morocco (*n* = 23), Peru (*n* = 7), an unspecified country (*n* = 1), and Venezuela (*n* = 34). Almost 43% of the sample came from Colombia. To simplify data analysis, they were grouped into two broader categories: Latin Americans (*n* = 103, 80.5%) and Africans (*n* = 25, 19.5%).

#### 2.2.4 Educational level

**Figure 1:**
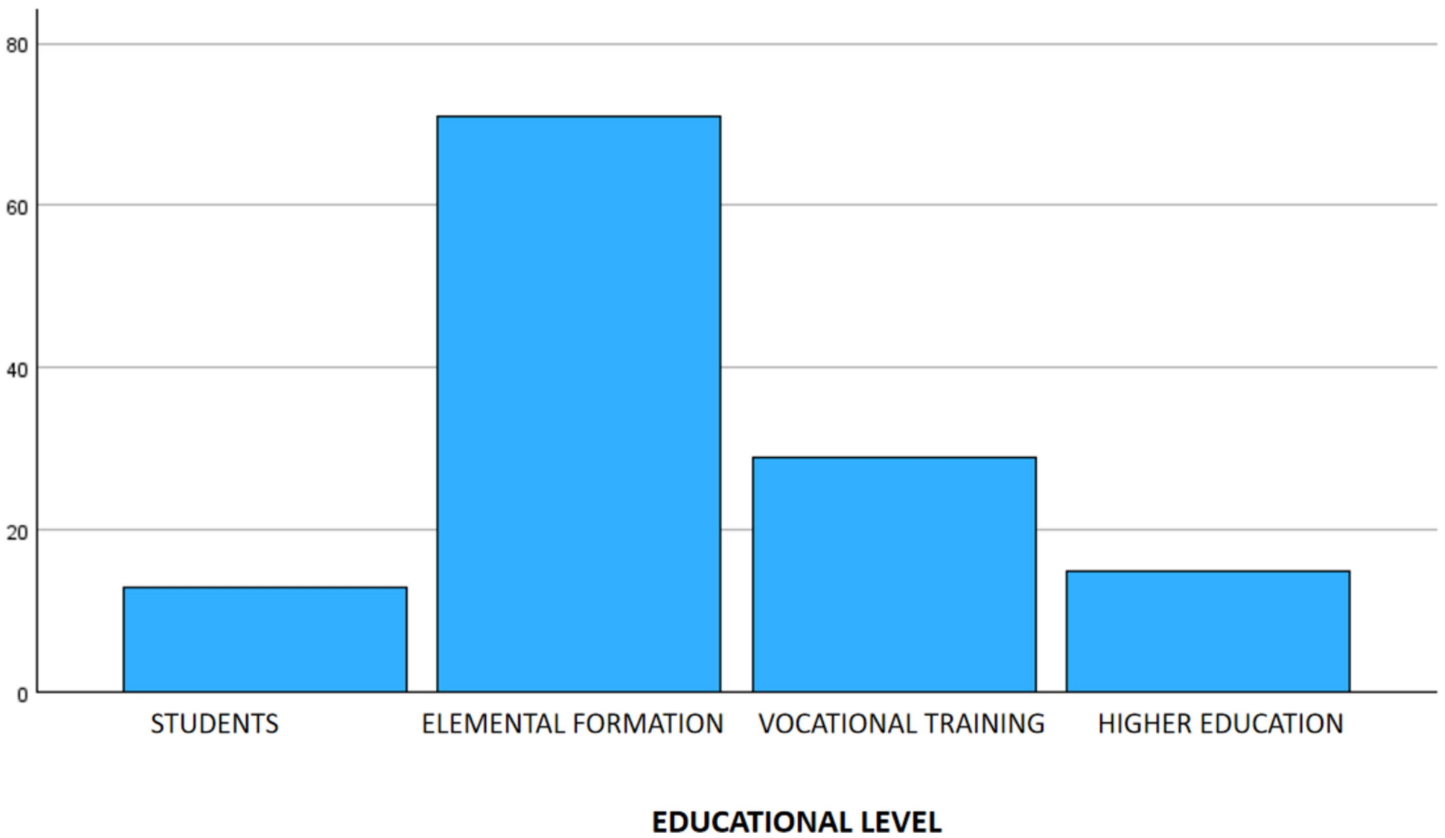
Distribution of the sample by educational level.

Participants reported a wide variety of previous occupations and education levels. For analysis, they were grouped as follows: Students (*n* = 13, 10.2%), Elemental Formation (*n* = 71, 55.5%), Vocational Training (*n* = 13, 10.2%), and Higher Education (*n* = 13, 10.2%) (Figure 1).

### 2.3 Statistical analysis

Quantitative variables were analyzed using the Student’s t-test, while qualitative variables were examined using the Chi-square test. A *p*-value of less than 0.05 was considered statistically significant. All statistical analyses were performed using SPSS version 29.0 (Statistical Package for Social Sciences, Chicago, IL, USA).

### 2.4 Ethical considerations of the study

This research involving human participants was conducted in accordance with the ethical standards of the institutional and/or national research committee, and with the 1964 Helsinki Declaration and its later amendments. Ethical approval was obtained from the Odontologia Social Foundation ethics committee (n° 04/24) approved in January 2024.

## 3 Results

### 3.1 Habits

#### 3.1.1 Sugar-sweetened beverage consumption

Eighty-five respondents (67.2%) consumed sugary drinks, while 42 (33.6%) did not. Among the 85 consumers, 69 drank one glass per day (80.2%), 7 drank two (8.1%), and 10 drank more than two (11.6%).

#### 3.1.2 Sugary food consumption

Eighty-two participants (64.1%) reported consuming sugary foods. However, this may be underreported due to the regular snack provided at the center.

#### 3.1.3 Toothbrushing frequency

Average of 2.88 times/day. Distribution: never (1.6%), once/day (11.7%), twice/day (36.7%), three times/day (50%). Gingival bleeding: 59.4% reported no bleeding; 40.6% reported bleeding during brushing.

#### 3.1.4 Smoking

**Figure 2:**
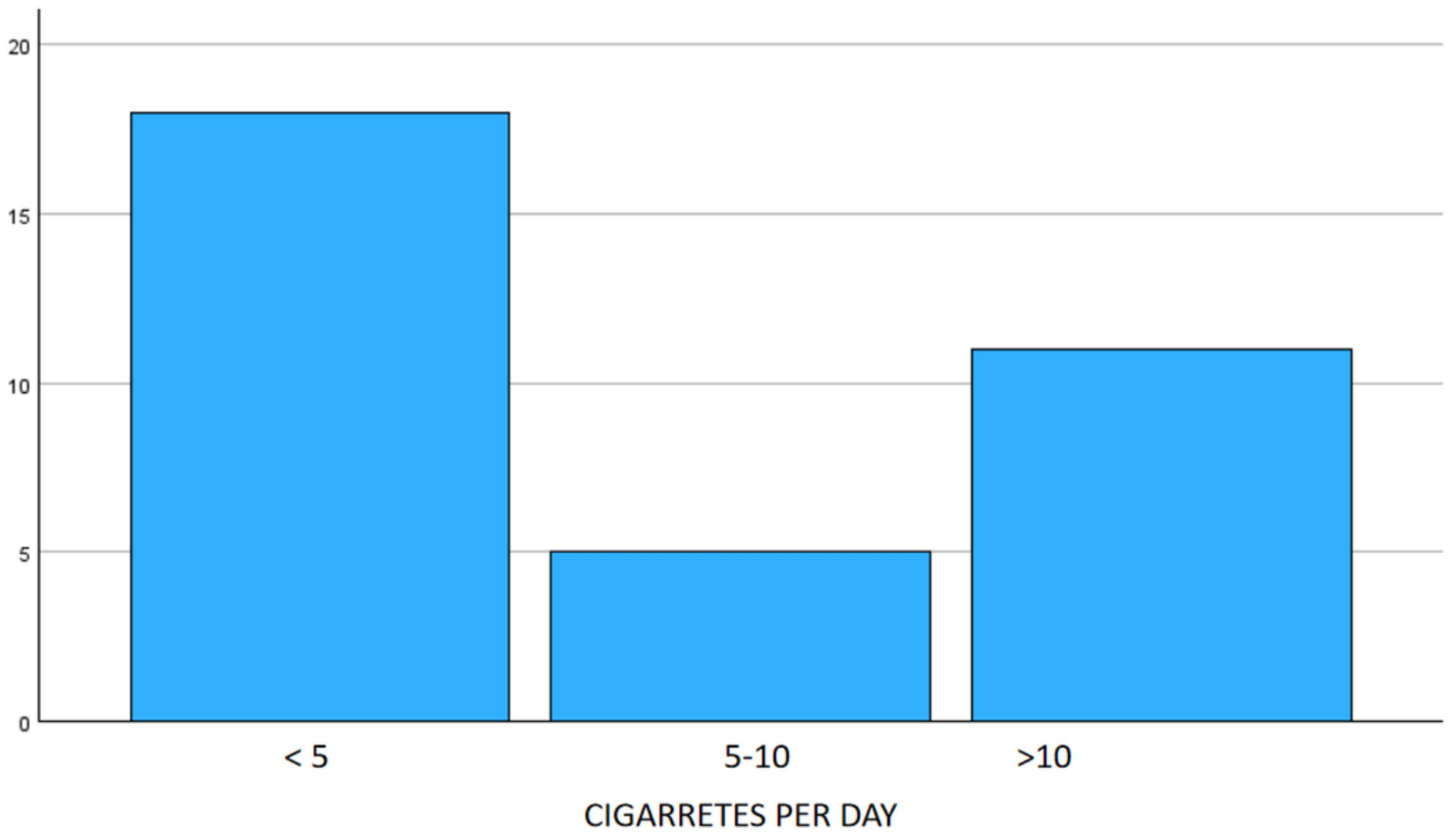
Smoking habits in the sample.

26.6% of respondents smoked. Among them, 52.9% smoked <5 cigarettes/day, 14.7% smoked 5–10 cigarretes, and 32.4% smoked >10 cigarretes (Figure 2).

### 3.2 Dental visits

Last dental visit distribution: <6 months (28.1%), 6 months–1 year (31.3%), 1–2 years (11.7%), 2–5 years (14.1%), >5 years (8.5%), never (6.3%) (Table 1).

**Table 1:** Reason for last dental visit.

Reason	Frequency	Percentage
Cannot remember	12	9.4%
Conservative treatment	29	22.7%
Orthodontic treatment	13	10.2%
Periodontal treatment	4	3.1%
Prosthetic treatment	5	3.9%
Emergency treatment	30	23.4%
Preventive treatment	35	27.3%

### 3.3 General oral condition

Dentition status: 32.9% fully dentate, 67.2% partially edentulous, 0% completely edentulous.

Use of dental prostheses: Only 9.4% wore partial dentures.

Mean values:

- Total teeth: 27.84.
- Healthy teeth: 24.15.
- Teeth with caries: 3.54.
- Filled teeth: 3.18.
- Missing teeth: 3.01.
- Missing due to caries: 1.57.
- Non-erupted: 1.14.
- Sealed: 0.12.

Tooth opacities: 90% had none. Others had diffuse or demarcated opacities, or hypoplasia.

Fluorosis: Absent in 97.7%.

Erosion: 91.4% had none. Minimal cases showed enamel, dentin, or pulp involvement.

Dental trauma: 93% had none. Minor cases of treated or untreated trauma observed.

DMFT Index: Mean value was 9.73 (range: 0–27).

### 3.4 Cross-tabulation of variables

As previously mentioned, in the analytical phase of the study, variables were cross-tabulated according to their qualitative and/or quantitative nature, and the corresponding statistical analyses were performed. A *p*-value of < 0.05 was considered statistically significant.

In this context, the chi-square test applied to qualitative variables revealed statistically significant associations between:

- Nationality and prosthesis-wearing status (*p* = 0.001).
- Nationality and smoking habits (*p* = 0.0042).
- Nationality and oral hygiene habits (*p* = 0.002).
- Gender and smoking habits (*p* = 0.009).

**Figure 3:**
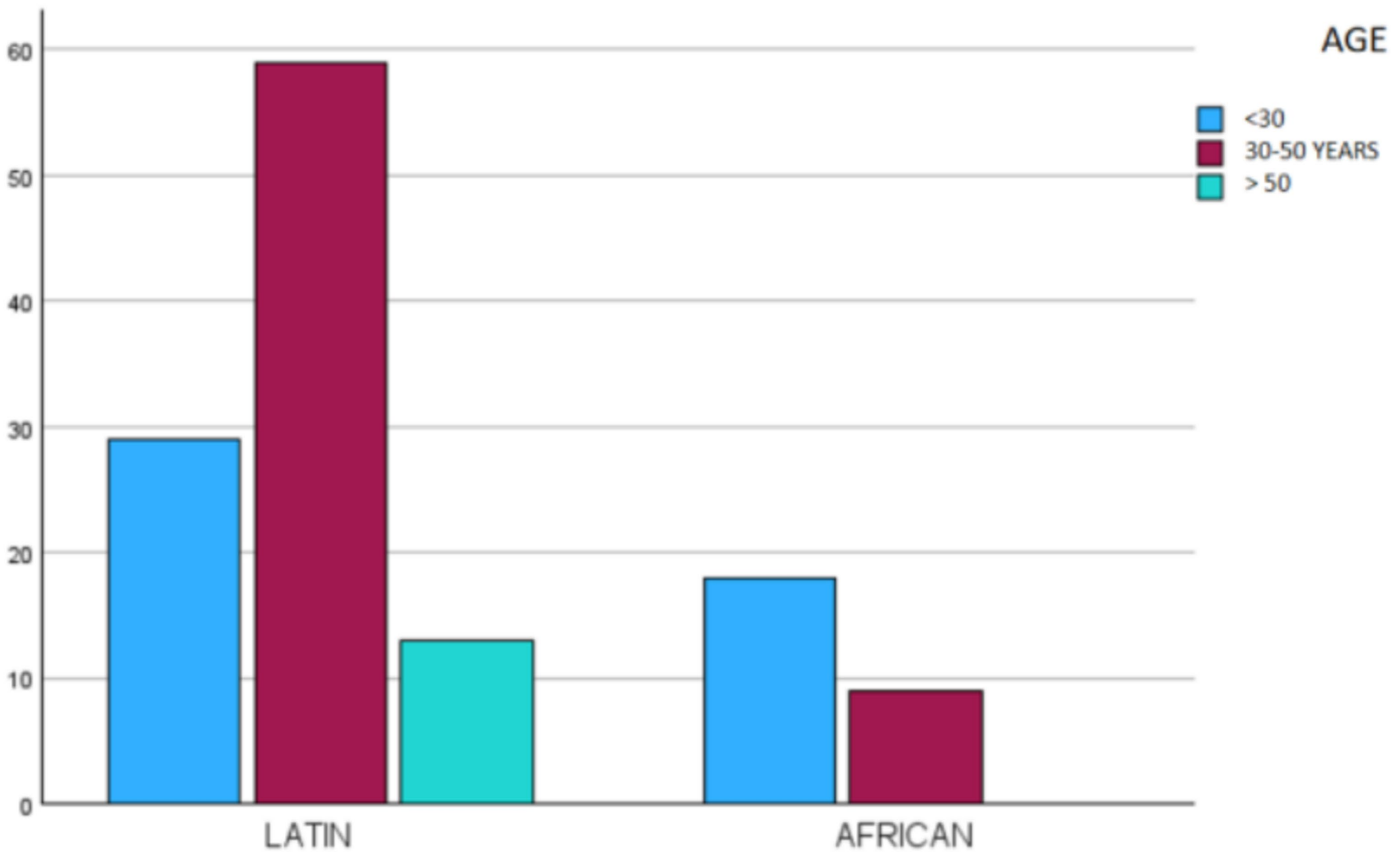
Distribution of the sample by age and place of origin.

When comparing origin with age groups, statistically significant differences were observed (Chi-square, *p* < 0.001). Latin American immigrants tended to be older, with greater representation in the 30–50 and 51–66 age groups, while African immigrants were mostly within the 18–30 age group and had no representation among older adults (see Figure 3).

## 4 Discussion

Migration is one of the most defining phenomena of our century. Population movements driven by inequalities between countries—and more recently, by political persecution or armed conflicts—have given rise to groups in extreme need and pockets of poverty and marginalization that receiving countries must manage and address. This study can only be understood within such a current and socially, economically, and culturally significant context as that of migration (12).

If we aim to design effective intervention strategies to improve the overall health—and, specifically, the oral health—of these populations, we must first understand their changing nature. For this reason, ongoing epidemiological studies such as this one are necessary and will continue to be essential. International migration patterns have shifted considerably in recent years, leading to a deeper understanding of the diversity among migrants—previously regarded as a fixed pattern tied to particular ethnic groups (21).

Focusing on our immigrant population at the CETI in Melilla, one of the first striking findings was the origin of the participants. Given the geographical location of the center, it would be expected that most of its users would be of African origin. However, of the 128 individuals surveyed, 103 (80.5%) were Latin American and only 25 (19.5%) were African. To understand this phenomenon, we must consider the history of the CETI, which, since its founding in 1999, has predominantly hosted African migrants.

Traditionally, refugees came from Algeria, Morocco, Mali, Guinea, Cameroon, Nigeria, Chad, Angola, Gabon, the Democratic Republic of the Congo, Niger, Zimbabwe, Ivory Coast, and many other sub-Saharan countries (25). In 2011, the Syrian civil war brought a large influx of Syrian refugees to the center (26). More recently, Latin American migrants have discovered that entering the CETI can help accelerate the bureaucratic process required to obtain residency in Spain. For this reason, many Latin American migrants already residing in the country in irregular situations are now arriving en masse, which explains the large number of Colombian and Venezuelan individuals among those surveyed (27).

The sex and age distributions found in our sample are consistent with the findings of other authors, with a higher proportion of young or middle-aged men and fewer women or older adult individuals (28). However, there was a statistically significant difference (Chi-square, *p* < 0.05) in the ages of migrants when comparing Latin Americans with Africans: the 30–50 age group was most frequent among Latin Americans, while the 18–29 age group was most frequent among Africans. Notably, there were no individuals over 50 among the African participants. This may be explained by the fact that many Latin Americans already have relatives established in Spain, which means that many respondents may be arriving to reunite with their families. Since almost all African migrants come from sub-Saharan countries, they must cross the entire Sahara Desert under harsh conditions to reach Melilla. This results in a migration composed of young people, which is why there are no individuals over the age of 50 of African origin in our sample (29).

One of the key issues to address is socio-health inclusion. Migration can lead individuals to lose access to even the most basic health care. In many cases, the harsh living conditions they are forced to endure significantly worsen their health status—including oral health—thereby hindering their integration into the host community. Temporary stay centers, such as the CETI, which provide individualized socio-health care, aim to reduce these disparities. As a result, migrants’ integration into receiving communities is improved, and potential rejection on health-related grounds is minimized (21).

With respect to oral health and associated behaviors, the data obtained in this study deviate from those of earlier research (25, 28, 29). Regarding diet, it is clear that sugar consumption is high. As explained in the results section, many participants may not be fully aware of their own consumption, introducing a bias in the survey results. Nonetheless, no significant differences were observed based on origin or gender.

The good oral hygiene reported by the participants is noteworthy, with an average brushing frequency of 2.35 times per day. This differs from previous reports by other authors (28, 30) and may be attributed to the origin of the surveyed populations. When comparing Latin Americans and Africans, statistically significant differences were found (Chi-square, *p* < 0.001) in favor of Latin Americans, who accounted for 80.9% of those brushing twice daily and 89.1% of those brushing three times daily. Notably, none of the Latin American respondents reported not brushing at all.

Another striking finding is that while 40.6% of participants reported gingival bleeding during brushing, only 3.1% reported having visited a dentist for periodontal treatment, and only 22% had sought restorative treatment. This is noteworthy considering the high prevalence of both pathologies according to the literature (15, 30). Gingival bleeding should be a clear indication for seeking dental care, as it may signal the presence of gingivitis or periodontitis—conditions that, if left untreated, can lead to tooth loss. Such a low rate (3.1%) of periodontal treatment suggests a lack of awareness, limited access, or low prioritization of oral health. The fact that only 22% of individuals had received restorative treatment further implies that many cases of dental caries are likely going untreated, thereby exacerbating long-term oral health issues. These findings may reflect a normalization of oral discomfort and a tendency to seek care only in acute or critical situations, while ignoring symptoms that should prompt preventive or early intervention.

The prevalence of smoking was not particularly high, with only 33% of participants reporting the habit, and among these, 53% smoked fewer than five cigarettes per day. However, there was a statistically significant difference between men and women, with a higher proportion of male smokers (Chi-square, *p* < 0.01).

Similarly surprising was the level of dental care received: nearly 60% of the surveyed individuals had visited the dentist within the last year, despite their status as migrants. Only 6.3% reported never having visited a dentist, and these were almost entirely of African origin. Differences in the time since the last dental visit were statistically significant between Latin Americans and Africans (Chi-square, *p* < 0.001), but not between men and women. Perhaps the lack of health awareness in this African population is the cause of this significant difference.

Another point of interest is the discrepancy between the need for prosthetic treatment and actual prosthesis use. Despite good hygiene habits and the relatively high number of teeth retained, 67% of the participants were partially edentulous, while only 9.4% used prosthetic devices.

Other dental health indicators—such as fluorosis, enamel opacities, erosion, or trauma—did not represent significant health problems due to their low prevalence.

The mean DMFT index (Decayed, Missing, and Filled Teeth) was 9.73, with values ranging from 0 to 27. This figure is considerably higher than that reported in the general population of the host country (19, 31). As this index explores the individuals’ history of dental caries, it is evident that the number of caries (treated or untreated) among the immigrant population is significantly higher than that of the host country’s population.

All of these epidemiological data—and those that may be gathered in future studies of immigrant populations—are essential for understanding these constantly evolving groups. They must be analyzed with the aim of reducing the health inequities, particularly in oral health, that these migrant populations face (10). This knowledge should also be complemented by a comprehensive understanding of the host country’s healthcare system and how to facilitate access to it for migrants, thereby promoting better integration and health outcomes (10).

## 5 Conclusion

This study reveals that the migrant population at the CETI in Melilla faces significant oral health challenges, including high rates of caries, tooth loss, and low use of dental prostheses, with notable differences by geographic origin. Despite generally adequate oral hygiene habits, poor oral health highlights gaps in access to preventive and therapeutic dental services.

Strengths include the use of standardized WHO methods and consideration of social determinants, allowing comprehensive analysis. Limitations involve a small sample size, cross-sectional design, and potential self-report bias. Health programs should focus on equity-based public health interventions, oral health education, improved access to dental care, and tailored treatments. Temporary centers can be key sites for preventive programs to reduce inequalities and support social integration.

Future research should include longitudinal studies to monitor oral health trends and qualitative studies on cultural, economic, and structural barriers. Intersectoral collaboration is essential for effective, sustainable strategies addressing the diverse needs of Melilla’s migrant population.

## Data Availability

The raw data supporting the conclusions of this article will be made available by the authors, without undue reservation.
