# Supplementary figures and images for: Social determinants of oral health in migrants at the Spanish border
Source: Front Public Health. 2025 Aug 1;13:1641311. doi: 10.3389/fpubh.2025.1641311 (PMC12354515; doi:10.3389/fpubh.2025.1641311)

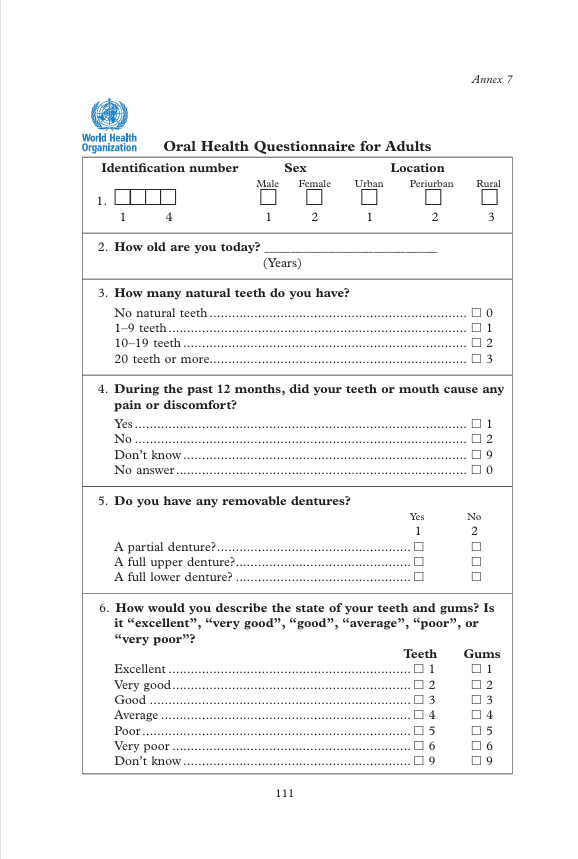


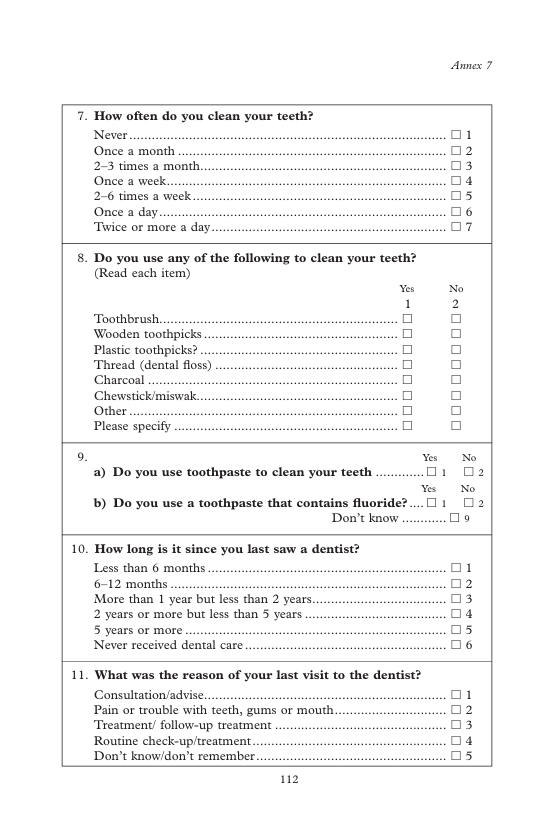


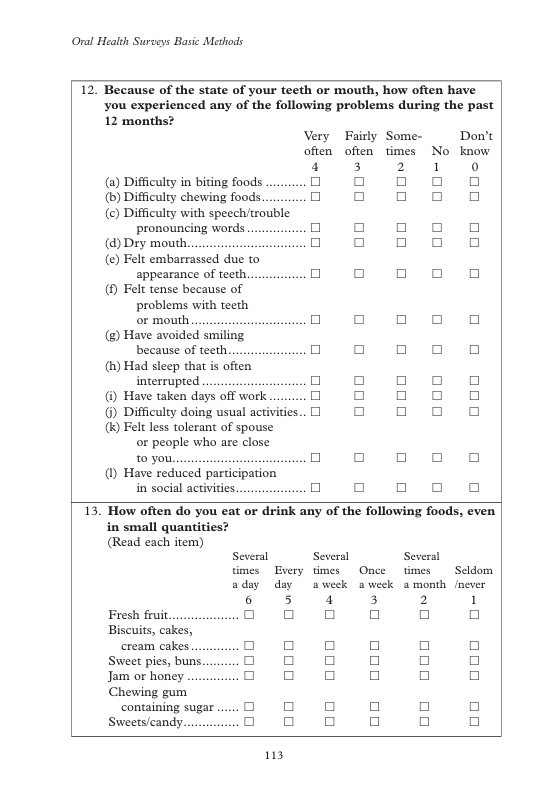


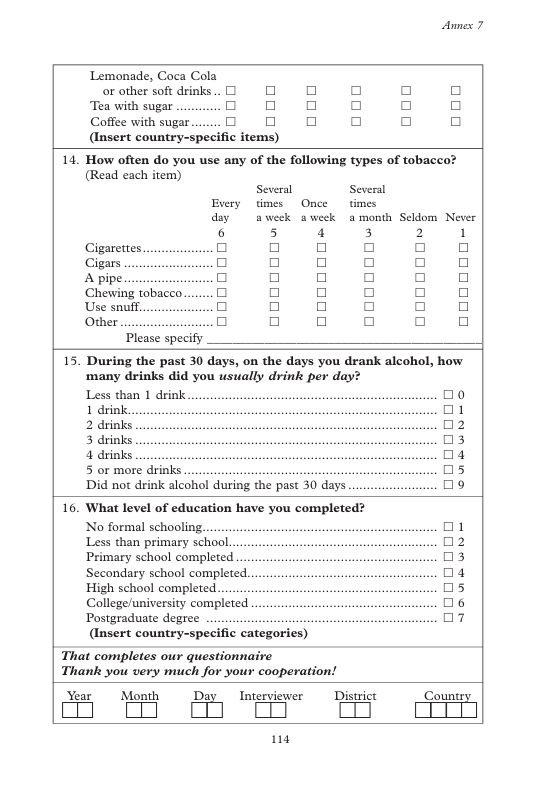

Supplement: Supplementary file 1 [file Table_1.docx]
